# Obesity and endoplasmic reticulum (ER) stresses

**DOI:** 10.3389/fimmu.2012.00240

**Published:** 2012-08-07

**Authors:** Yamini B. Tripathi, Vivek Pandey

**Affiliations:** Department of Medicinal Chemistry, Institute of Medical Sciences, Banaras Hindu UniversityVaranasi, India

**Keywords:** ER stress, mitochondrial stress, obesity, inflammation, metabolic syndrome

## Abstract

In obesity, the adipose cells behave as inflammatory source and result to low grade inflammation. This systemic inflammation along with oxidative stress is a silent killer and damages other vital organs also. High metabolic process, induced due to high nutritional intake, results to endoplasmic reticulum (ER) stress and mitochondrial stress. This review describes the triggering factor and basic mechanism behind the obesity mediated these stresses in relation to inflammation. Efforts have been made to describe the effect-response cycle between adipocytes and non-adipocyte cells with reference to metabolic syndrome (MS).

## Introduction

**Figure 1:**
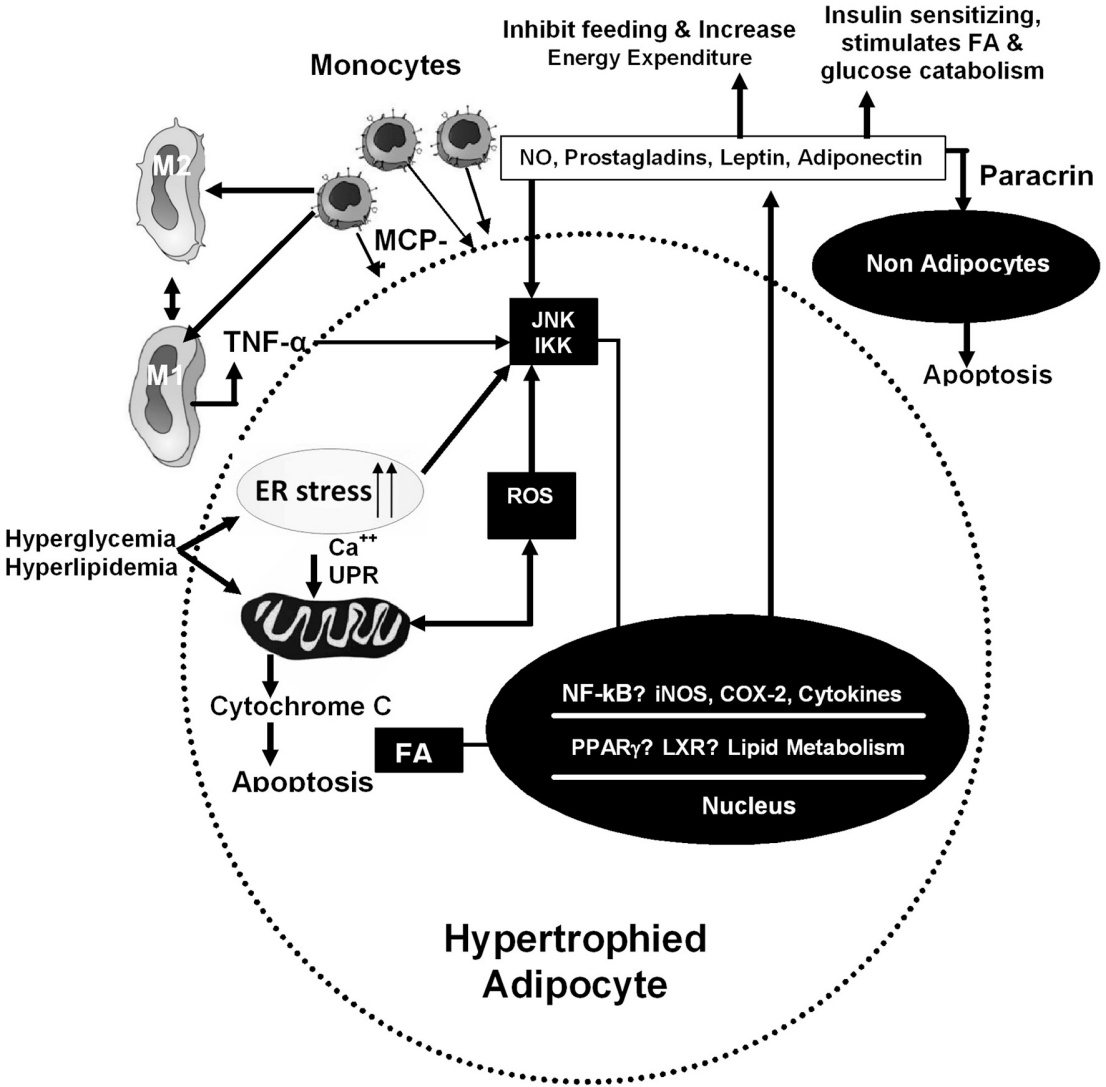
**Inflammatory and metabolic pathways in adipocytes.** These pathways are induced by intracellular and extra-cellular signals. In hypertrophied adipocytes, infiltration of macrophages increases, resulting to increased expression of genes involved in inflammatory cascade. On hyperphagia, ER stress increases in cells which is linked with mitochondrial dysfunction and release of cytokines.

Increased body mass index (BMI) is associated with metabolic syndrome (MS), a cluster of central obesity, insulin resistance, impaired glucose tolerance, hypertension, atherosclerosis, and dyslipidemia. It is accompanied with low grade inflammation, vascular endothelial dysfunction, high adipokines, hypoxia, oxidative, and metabolic stress. In obesity, the adipose cells behave as inflammatory source and result to systemic inflammation. High caloric nutrition, oxidative stress, and dyslipidaemia are the basic factors, resulting to high circulating FFA, insulin resistance, and macrophage dysfunction. The basic cause is the behavioral change in adipocytes, which is primarily mediated through ER dysfunction and further linked to oxidative stress and mitochondrial dysfunction (Figure 1). The whole process changes the phenotype of macrophages, through its polarized differentiation, ultimately setting up of low grade inflammation. Here, we have discussed the initiating causes of fat deposition in adipose and non-adipose tissue and their changed behavior; identification of molecules, responsible to induce ER stress; the pathway to link ER stress with mitochondrial dysfunction and polarized differentiation of monocyte derived macrophages. The possible cause–effect relationship between low grade inflammation and initiation of MS has also been discussed.

## High fat accumulation in adipocytes and non adipose tissues

The body has inherent capacity to store extra fuel for starvation driven emergency. The fat is the storage form of extra nutritional calories and the adipose cells are the primary site for its deposition. However, after fat-overloading, the extra fat overflows from these cells and get their resting destination to non-adipocytes. This phenomenon is called ectopic fat storage. Although, fat is deposited in all kind of tissues, but muscle and liver are the main organs. During its transport and also in tissue, the scavenger macrophages keep on engulfing the fat droplets, to maintain the normal level as high FFA is liable to disturb the homeostasis of the tissue. Finally, these macrophages get converted to foam cells. The oxidized and glycated lipoproteins, free fatty acids, free cholesterol, triacylglycerols, diacylglycerols, and ceramides are predominant lipid intermediates, which deposit to these organs and induce cellular dysfunction. In case of diabetes, high diacylglycerols activates PKC, which further activates serine/threonine kinases and inhibits insulin signaling (Bergman et al., 2012).

In case of normal fat deposition, peripheral and visceral adipocytes are the primary targets. The preference to these tissues is genetically governed as Asian population is more prone to visceral fat deposition. In fact, this is a kind of adaptive process to reduce the circulation free fatty acid in the body, which is more harmful. It acts as the reservoir to trap high circulating free fatty acid in the body. It is a mechanism to prevent ectopic fat deposition, onset of fatty liver and insulin resistance. Among, these two tissues also visceral fat is considered to be more harmful than peripheral fat, because of its higher inflammatory nature, as described below (Tsuriya et al., 2011).

The basic cause of inflammatory nature of these adipose tissues is to due to high accumulation of tissue imbedded macrophages and also because of its own cellular stresses. A report indicates that when mice are given a restricted diet, macrophage recruitment is unexpectedly increased in adipose tissue during the early phase of weight loss (Kosteli et al., 2010). Thus macrophages could contribute to ensure adipose tissue homeostasis and remodeling in response to changes in energy balance and body weight alterations. They display phagocytic activity against lipid droplets, without causing inflammation, thereby contributing to restore local lipid homeostasis. Thus, macrophage accumulation is a kind of adaptive change. An increase in macrophage number is concomitant with increased lipolysis in adipose tissue and is not associated with up-regulation of inflammatory genes.

## Adipocytes and inflammation

The inflammatory role of adipocytes is related to its expansion, hyperplasia and hypertrophy. They involve variety of cellular stresses like endoplasmic reticulum (ER) stress, mitochondrial dysfunction, oxidative stress etc. These factors change the secretion property of adipocytes, which can be assessed through altered secretion profile of adipokines. They act locally (autocrine/paracrine) and also systemically (endocrine). In normal conditions, there are three main adipokines, i.e., (a) leptin, (b) adiponectin, and (c) resistin, but in fat-overloading, these adipocytes secrete additional inflammatory cytokines and free fatty acid. They express novel receptors for various endocrine and neural signals (Kershaw and Flier, 2004). They attract more macrophages (Weisberg et al., 2003), which get imbedded into adipose tissues. Thus, cluster of macrophages with adipocytes are form a crown-like structures (CLSs) which are more inflammatory.

The Adipocytes hypertrophy behaves as the source and target of inflammatory signals. It induces expression of TNF-α, IL-6, IL-1β, PG-E2, etc (Apovian et al., 2008). These adipocytes secrete some novel cytokines, which convert a subset of circulating monocyte to macrophages (Ronti et al., 2006). There is low secretion of adiponectin, which enhances the process of hepatic necrosis and inflammation. Eventually, this hypertrophy leads to adipocyte's death and the release of cellular contents into the extracellular space, which further attracts macrophages (Cinti et al., 2005) and persistent activation of neutrophils, monocytes, and T-cells.

In obesity, the plasma level of C-reactive protein (CRP) is high, which is largely synthesized by the liver. Its synthesis is enhanced in response to all inflammatory cytokines, described above. The CRP further amplifies the pro-inflammatory effects of other adipokines. Besides inflammation, the adipocytes also possess oxidative stress and release heme oxygenase-1 (ho-1) and show low plasma superoxide dismutase (SOD) activity. The oxidative stress is associated with high hydrogen peroxide (H_2_O_2_) accumulation and high expression of NADPH oxidase-IV. At later stage, high urinary TBARS and change in urinary pH have been noted. The ER also contributes to oxidative stress by free radical generation. It is through activation of protein disulfide isomerase (PDI), which catalyzes disulfide bridge formation (Schroder and Kaufman, 2005).

In various reports, it was suggested that as adipose tissue mass expands in obesity, clusters of enlarged adipocytes become distant from the vasculature, leading to local pockets of hypoxia (Trayhurn and Wood, 2004). Hypoxia-inducible factor-1α (HIF-1α) is induced in human adipocytes by hypoxia and is regarded as “a master hypoxia signal mediator” (Semenza, 2002; Wang et al., 2007). Hypoxia in turn induces the gene expression of inflammatory markers in adipocytes and macrophages. The induced genes include TNF-α, interleukins (IL)-1, IL-6, monocyte chemoattractant protein-1 (MCP-1), and plasminogen activator inhibitor-1 (PAI-1).

## Obesity and macrophage infiltration

The adipose tissues in obese persons are associated with raised leptin (Levin, 2005) and low adiponectin in plasma (Bouhali et al., 2008). Enlarged adipose tissue recruits macrophages and lymphocytes (Kewalramani et al., 2011). Later on they undergo apoptotic process and also release inflammatory cytokines and chemo-attractants. Increased accumulation of macrophages is due to influx of its bone marrow–derived precursors to adipose tissue and their subsequent differentiation into mature F4/80-expressing macrophages. The presence of CD68-positive macrophages in direct contact with mature adipocytes has been noted more in visceral fat than in subcutaneous peripheral fat (Levin, 2005).

Release of MCP-1 and macrophage colony stimulating factor (M-CSF) are major mediators for macrophage accumulation. Transgenic expression of MCP-1 also known as chemokine ligand 2 (Ccl2), in adipose tissue increases macrophage infiltration, inflammation, and insulin resistance (Kaufman, 1999; Munzberg and Myers, 2005; Brennan et al., 2007). Conversely, disruption of MCP-1 or its receptor CCR-2 impairs migration of macrophages into adipose tissue, thereby lowering adipose tissue inflammation and improving insulin sensitivity (Baskin et al., 1999; Kaufman, 1999).

At one hand adipocytes undergo ER stress due to high lipid accumulation and metabolism, thereby some of them are undergoing apoptosis, but on the other hand, the tissue imbedded macrophages trap the fat droplets and also engulf the dead adipocytes through phagocytes. This process is associated with respiratory burst and release of high amount of free radicals. This high volume of reactive oxygen species (ROS) further enhances the mitochondrial dysfunction, ER stress, and cytoplasmic pH change. All these factors induce a cellular stress, which changes the phenotype of adipocytes, resulting to high secretion of adipokines. At paracrine level, these secretions alter the macrophage-phenotype. They undergo polarized differentiation between pro-inflammatory M1 type (classically activated macrophages) and non-inflammatory M2 type (alternative activated macrophages) (Figure 1). The expression of CD36 in both macrophages and adipocytes, enhances adipose tissue inflammation and cell death in diet-induced obesity.

The phenotypic change in macrophages, between M1 and M2, differs in obese and lean animals. It is predominantly dependent on the local cytokine released in that tissue, where these macrophages are embedded. In lean mice, the imbedded macrophages have high expression of F4/80^+^CD11c^+^ proteins, characteristic of M2 macrophages. However, in obese mice, these expressions are low and here M1 macrophages are predominant. They are inflammatory in nature and has characteristic gene expression of TNF-α and iNOS, IL-1β, CXCL10, and IL-6. The M1 macrophages limit the adipocyte hypertrophy (Lumeng et al., 2007) and inhibit adipogenesis and increase the extracellular matrix deposition in pre-adipocytes (Keophiphath et al., 2009). Hence, inhibition of adipogenesis combined with a pro-fibrotic phenotype of pre-adipocytes strengthens the role of pro-inflammatory macrophages to restrain adipocyte size. Because of local release of cytokines by adipocytes, large numbers of immune cells, including monocytes and T-lymphocytes, are aggregated in adipose tissue (Moller and Kaufman, 2005).

Macrophages are classically stimulated by interferon-γ (IFN-γ) alone or in combination with lipopolysaccharide (LPS) and they produce inflammatory cytokines (e.g., IL-1, IL-6, and TNF-α), which are capable of inducing Th1-polarized T-cell responses. The adipocytes, undergoing necrosis, secondary to hypertrophy, also lead to macrophage activation (with the accompanying release of inflammatory mediators) and their subsequent clearance from adipose tissue (Levin, 2005). It may lead to systemic insulin resistance, as reported in diet-induced animal model (Alkhouri et al., 2010; Kanda et al., 2006).

## Endoplasmic reticulum (ER) stress

The ER is a critical site of protein, lipid and glucose metabolism, lipoprotein secretion, and calcium homeostasis. It is mainly recognized as a protein-folding factory and involved in lipid synthesis, protein folding, and maturation. It also helps in biosynthesis, folding, and modification of numerous soluble and membrane bound proteins (Kaufman, 1999). Most of the newly synthesized proteins are transported to the lumen of the ER, for proper folding for their target places in the cell. Many signal sequences define the pathway of Proteins transport from ER to the cell. A few amino acids of N-terminus work as an address tag, which are removed when the polypeptide reaches to its destination. Proteins that are destined for places outside the ER are packed into transport vesicles and moved along the cytoskeleton toward their destination. The smooth endoplasmic reticulum (SER) has many functions as, synthesis of steroids, metabolism of carbohydrates, regulation of calcium concentration, drug detoxification, attachment of receptors on cell membrane proteins, and steroid metabolism (Tsuriya et al., 2011).

Thus ER stress is referred as a condition, where accumulation of unfolded or misfolded proteins in ER lumen. This is called Unfolded Protein Response (UPR). The improper-folded-proteins, released due to ER stress, do not reach to Golgi apparatus, but released in cytosol, for their degradation by proteosomes. The N-glycosylated proteins in the ER regulate the folding quality. Genetic over expression of the ER chaperones ORP150 (oxygen-regulated protein-150) and GRP78 (glucose-regulated protein-78) improves metabolic regulation in mice. Moreover, treatment of insulin-resistant humans with TUDCA, a conjugated bile acid derivative that inhibits ER stress—induced apoptosis, results in increased insulin sensitivity (Kars et al., 2010). The observation that treatment with chemical ER chaperones reduces both obesity/insulin resistance and atherosclerosis in mice further supports this conclusion. ER stress transcript HSP-A5, which is high with increased BMI (Wang et al., 2009).

ER stress in adipose tissue may be due to nutrient over-load, along with increased demand for protein synthesis for its metabolism, local glucose deprivation due to insulin resistance, and decreased vascularization. There is excessive protein traffic or by excess accumulation of unfolded protein aggregates, collectively known as the UPR. It is also an adaptive step but high UPR initiates inflammation (Achard and Laybutt, 2012). High-fat diet also induces ER stress in other tissues also. It has been linked to ER stress in the hypothalamus, through high protein kinase-like ER kinase (PERK) phosphorylation (Özcan et al., 2004). In turn, it leads to insulin resistance and Type 2 diabetes (Harding et al., 2001). Here, high FFA also down regulates PPARγ protein and its mRNA (Haukeland et al., 2012). ER stress can be measured through other key indicators such as HSPA5 (heat shock protein-A-5), CHOP (C/EBT-homologous Protein), ERN1, and GADD34. There are other ER stress marker genes also e.g., Atf4, activating transcription factor 6 (Atf6), Xbp1s, and inositol-requiring protein-1α phosphorylation as well as changes in eIF2α phosphorylation (Boyce et al., 2005). Besides, these UPR markers, it has been reported that ATF6α expression is reduced in the liver of obese mice under ER stress. Its reconstitution through adenoviral-ATF6α, improves glucose homeostasis in these animals (Zhang et al., 2002). This suggests a possible dissociation between chaperone production to enhance protein processing and other UPR-mediated signaling events that could underlie some of the detrimental metabolic effects seen in obesity. Hence, a mild balance between glucose levels and the UPR needs to be maintained.

Similarly, cholesterol induces ER stress in liver, via high activity of Lecithin:Cholesterol acyltransferase (Hager et al., 2012). Cholesterol, either synthesized in the ER or derived from the diet, contributes to cell membranes. The whole process of cholesterol trafficking involves ER activation. There is high release of ER calcium and ROS generation in cholesterol overloaded macrophage (Pahl and Baeuerle, 1997). Here, three ER stress sensors are activated e.g., PERK, IRE1α, and ATF6, along with their downstream effectors-molecules. The overload with unesterified cholesterol in the ER membranes of macrophages decreases the calcium store of ER (Feng et al., 2003), resulting to ER stress and UPR. They form the foam cells, which are inflammatory in nature and induce the synthesis of pro-inflammatory cytokines (TNFα and IL6), by activating the nuclear factor kappa B (NF-kB) and the mitogen-activated protein kinases-mediated inflammatory pathways (Li et al., 2005). Here, CHOP, the target of PERK-mediated UPR pathway, is required (Li et al., 2005).

High activity of lipoprotein lipase (LPL) also initiates ER stress. Diets high in refined carbohydrates have been shown to cause tissue-specific over-expression of LPL through hyper-insulinemia (Johansen and Malmlöf, 2006). The adipocyte-LPL is activated by insulin and muscle LPL is activated by glucagon and epinepherine. Therefore, fasting enhances LPL activity in muscle and decreases in adipose tissue. This response is opposite to well fed individuals. Drugs like thiazolidinedione (TZD) increases systemic insulin sensitivity and adipose tissue triglyceride storage while decreasing its fatty acid efflux. Insulin activates LPL in adipose tissue but decreases in muscle (Shearer et al., 2011).

The ER of adipocytes plays a major role in the assembly and secretion of adipokines. The ER stress significantly decreases the adiponectin mRNA expression. Further ER stress also induces “leptin resistance.” High homocysteine level also induces ER stress (Kokame et al., 2000; Zhang et al., 2008), resulting to leptin resistance (Delépine et al., 2000). Thus all these phenomena lead to insulin resistance.

It has been reported that SOCS-3 (suppressor of cytokine signaling-3) (Cheng et al., 2002; Zabolotny et al., 2002) or PTP-1B (protein tyrosine phosphatase-1B) (Outinen et al., 1999; Hosoi et al., 2008) are involved in leptin resistance. In liver, ER stress increases expression of hepatic gluconeogenic enzyme. This pathway is also involved in suppression of hepatic gluconeogenic enzymes, which is via JAK2 dephosphorylation and HDAC-dependent STAT3 deacetylation. Thus, it increases the hepatic glucose production in obesity and diabetes (Kimura et al., 2012). The fatty acid-binding proteins (FABPs), especially FABP4 and FABP5, a family of lipid chaperones, play significant roles in promotion of metabolically triggered inflammation known as metaflammation in MS linked diseases, which is high in ER stress.

The ER stress is also found in hyperglycemia. It is because of high insulin demand. It allows the entry of new pro-insulin into the ER for maturation. On continuous increase of blood glucose, pro-insulin translation is raised along with high UPR. This results to activation of secretion capacity of the ER for active insulin (Scheuner et al., 2005). Since, these cells are very rich in ER network, because of high turnover of insulin protein in this organ, so ER stress significantly compromises its function. In pancreatic β cells, low blood glucose activates PERK mediated UPR pathway, due to low energy for protein folding in the ER. This results to translational attenuation to reduce the ER workload. When blood glucose levels increases, the UPR pathways are deactivated, resulting to accelerated translation. It is reported that pre-existence of mild ER stress, predisposes β-cells to an exacerbated inflammatory response when exposed to IL-1β or TNF-α, cytokines that contribute to the pathogenesis of type 1 diabetes (de Oliveira et al., 2012). These cells show more intense and protracted inflammatory response through inositol-requiring enzyme 1/XBP1 activation (Miani et al., 2012).

There is suppression of protein synthesis and stimulation of lipid synthesis in the ER of obese animals, without significant alterations in chaperone content. The ER chaperone proteins play important role in correct folding of newly synthesized protein. These chaperones are disulfide isomerase (PDI), ERp29, the Hsp70 family member Grp78, calnexin, calreticulin, and the peptidyl propyl isomerase family. ER stress is suggested to be the proximal cause of inflammation in adipocytes, PTP1B expression levels, involved in the ER stress response, are increased in adipose tissue of obese, high-fat-diet-fed mice (Shearer et al., 2011). The ER is also involved in xenobiotic detoxification and dynamic calcium storage. The activation of rapamycin activity is another condition leading to ER stress. It is highly activated in obesity, especially in metabolically active tissues like liver, adipose tissue, and pancreatic islets (Ozcan et al., 2006).

ER stress is associated with inflammation. The 12/15-lipoxygenase enzyme (12/15-LO) mediated pathway has been reported to be disturbed in adipocytes, pancreatic islets, and liver cells during ER stress. It promotes inflammation and insulin resistance in adipose and peripheral tissues. It acts through high expression of BiP, XBP-1, p-PERK, and p-IRE1α, which are similar to expression induced by tunicamycin or thapsigargin, a known ER stress inducer (Cole et al., 2012).

The induction of inflammation by independent Toll-like receptor 4/2-pathway has been reported due to ER stress, induced by intracellular accumulation of stearic acid. It finally induces apoptosis in adipocytes. It is via activation of JNK pathway and depletion in ER Ca^++^. Use of its inhibitors has reduced the apoptosis in adipocytes. Further, blocking the transcription factor CHOP also delays such apoptosis. Alterations in ER fatty acid and lipid composition inhibit the sarco/ER calcium ATPase (SERCA) activity resulting to ER stress. Here, ER stress sensors IRE1 (the inositol-requiring ER-to-nucleus signal kinase 1), PERK, and ATF6 are activated, resulting to reduction of translation and increase in transcription of ER chaperones to ensure the normal cell function.

The ER stress is also associated with dysfunction in the exocrine pancreas, resulting to diabetes (Delépine et al., 2000). Moreover, hyperglycaemia in the diabetic Akita mouse was found to be accompanied by CHOP expression and the apoptosis of β-cells. In addition, the deletion of CHOP has been shown to reduce oxidative stress, improve β-cell function and promote cell survival in multiple mouse models of diabetes (Xu et al., 2003). These results suggest that ER stress is involved in diabetes and that several ER-stress-related genes are involved in these processes.

There is direct or indirect induction of ER stress response by advanced glycation end products (AGEs). The advanced glycation is the major posttranslational modification of proteins, DNA, and lipids. It is accelerated under conditions of increased oxidative stress, hyperglycemia, and hypoxia. It contributes to a variety of metabolic diseases such as diabetes mellitus, obesity, inflammation, polycystic ovarian syndrome, ischemic cardiovascular disease, and neurodegenerative disorders. Inhibitors of advanced glycation, acting as potent ER stress modulators, show beneficial effects and restores ER homeostasis. It is emerging approach to treat MS. Thus, a crosstalk between Advanced Glycation and ER Stress has been reported (Piperi et al., 2012).

The ER-stress also induces autophagy in adipocytes. It is another way for manifestation of obesity-induced insulin resistance and type-2 diabetes. The UPR activates the ER-associated degradation (ERAD) system so that miss-folded/unfolded proteins get transported from the ER lumen to the cytosol for subsequent degradation, through autophagy-dependent pathway. Autophagy is a pathway for cellular defense mechanism, where recycling of nutrients and energy takes place for cell survival. During autophagy, cytoplasmic constituents are sequestered into double membrane vesicles (autophagosomes) that subsequently fuse with lysosomes for degradation (Zhou and Liu, 2010).

## Interaction between ER stress and mitochondrial dysfunction

The ER oxidative stress. It generates ROS, via activation of PDI, an enzyme which catalyzes disulfide bridge formation and in the process generates ROS (Cinti et al., 2005). ER stress is linked to mitochondrial dysfunction through modulated Ca^2+^ signaling (Szabadkai and Duchen, 2008). The releases of Ca^2+^ from the ER lumen, leads to increased Ca^2+^ uptake by mitochondrial matrix. It induces an imbalance between mitochondrial Ca^2+^ load and the buffer concentration of the matrix. Such imbalance causes a prolonged mitochondrial Ca^2+^ accumulation and opening of mitochondrial permeability transition pore (mtPTP). It finally causes mitochondrial swelling, breaking of the OMM, and release of pro-apoptotic proteins into the cytoplasm (Deniaud et al., 2008).

During excess fat/carbohydrate intake, adipocytes initiate the process of glyceroneogenic and lipogenic steps at higher rate. This is supported by supplies of its precursors e.g., pyruvate, lactate, and alanine, which involves high activity in mitochondria through activation of pyruvate carboxylase. ATP, reducing cofactors and Acetyl-CoA are the obligatory cofactor for these pathways (Yen et al., 1977). The availability of ATP directly regulates the rate of lipogenesis in adipose tissue is the ATP availability as One acetyl-CoA moiety formed from pyruvate and incorporated into fatty acids requires three molecules of ATP. Thus, ATP depletion may retard lipogenesis. Contrary to this ADP inhibits this process thus ATP:ADP ratio is important. The activity of pyruvate carboxylase in mitochondria of rat adipose tissue is greater than the concentration, found in mitochondria of liver. This whole process imparts a kind of stress to mitochondria, which produces more superoxides as byproduct. Thus a kind of oxidative stress is generated within the cells of adipose tissue, which further initiates ER stress and inflammatory process.

The ER stress mediated mitochondrial dysfunction is also mediated by nitric oxide (NO) production (Xu et al., 2004). In competition with oxygen, NO can bind to cytochrome C oxidase and inhibits its activity (Brown, 2001). It changes the Ca^2+^ movement between mitochondria and ER. Further the NO mediated changes in Ca^2+^ flux, increases expression of ER stress responsive genes such as glucose regulated protein-78 (Grp-78). Similarly, the mitochondrial dysfunction in muscle is associated with the onset of insulin resistance and type 2 diabetes.

Cysteine is the most reactive amino acid in protein and wide ranges of cysteine derivatives are formed *in vivo*, resulting from oxidation, nitrosation, alkylation, and acylation reactions. Similarly, succination of proteins takes place, which is an irreversible chemical modification of cysteine by the Krebs cycle intermediate, fumarate, and yielding S-(2-succinyl) cysteine (2SC). It is increased due to hyperpolarization of the inner mitochondrial membrane and develops mitochondrial and oxidative stress. The succination of glyceraldehyde-3-phosphate dehydrogenase has been found to be raised in the muscle of streptozotocin-diabetic rats. There is also increased succination of adiponectin, which results to decreased secretion of adiponectin from adipose tissue in type-2 diabetes. Thus, 2SC serves as a biomarker of mitochondrial stress. It serves as the mechanistic link between mitochondrial and ER stress in diabetes.

Contrary to above facts, mitochondrial dysfunction also induces ER stress. Since, large amount of ATP is required for both protein folding and handling of Ca^2+^ within the ER, so in its scarcity ER stress is induced (Kaufman, 2002). The inhibition of oxidative phosphorylation causes delay in uptake of Ca^2+^ into the ER-lumen. This low level of Ca^2+^ within the ER inhibits the oxidative phosphorylation, resulting to rapid ATP depletion in mitochondria and the ER. When this condition is prolonged, the cytosolic ATP level decreases. Some studies show that ROS acts as a local messenger between ER and mitochondria (Csordás and Hajnóczky, 2009). Large number of ROS sources and targets are located in ER and mitochondria (Malhotra and Kaufman, 2007). In folding of newly synthesized ER, the oxidoreductin 1 (Ero1) protein-family mediated formation of disulfide bond is a critical step, which is activated in ER stress (Harding et al., 2003). Further, due to excessive production of ROS in the ER, the Ca^2+^ ATPase (SERCA) of sarco-ER gets inactivated and IP_3_R gets activated (Adachi et al., 2004). The ROS also changes Ca^2+^ channel activity of ER, which increases the level of Ca^2+^ on the cytosolic face of the ER. Thus ROS mediated Ero1 expression provides an additional mechanism by which ER stress induces mitochondrial dysfunction.

## Therapeutic target and therapies for management of cellular stresses

Several studies have targeted signals involved in the inflammatory process of obesity. It includes inhibition of expression of inflammatory cytokines, modulation of ER stress and mitochondrial stress, apoptotic process mediated signals in adipocytes, infiltration and phenotypic modification of adipose tissue macrophages, liver function, with reference to visceral fat accumulation etc., the steps involved for augmentation of the process of protein folding, modulation of protein chaperones to curve down the production of abnormal proteins and UPR. Down regulation of Ca^++^ release from ER and attenuation of ER protein synthesis, through inhibition of eIF2alpha dephosphorylation are other approaches. It decreases the ER protein translation and reduces apoptosis (Gregor and Hotamisligil, 2007).

The PPAR-γ is a target, because its activation decreases the size of adipocyte and improves the insulin sensitivity on skeletal muscle (Hsueh and Bruemmer, 2004). It also decreases ER protein synthesis by increasing eIF2alpha phosphorylation. Some PPAR-γ agonists increase the uptake of free fatty acids into the fat cell as they promote fat cell differentiation and decrease inflammatory markers. They also increase adiponectin expression (Ofei et al., 1996; Hsueh and Bruemmer, 2004).

The neutralization of TNF-α in obese rodent IS reported to improve the glycemic control and dyslipidemia (Ma et al., 2004). Plasminogen activator inhibitor 1 (PAI-1) the most important inhibitor of fibrinolysis, is synthesized predominantly in vascular tissues, liver, and visceral adipose tissue. The PAI-1-deficient mice are protected against insulin resistance and obesity. The antagonists of angiotensin AT1 receptors, down regulate PAI-1 and improves diet-induced obesity and hyperglycemia in mice (Williams, 2012). Thus, regulators of PAI-1, which include regulators of the renin–angiotensin system (RAS), might have beneficial effects in the prevention and treatment of cardiometabolic disease. Thus, drugs, which induce LPL expression in non-hepatic tissue, may be helpful in its management.

The treatment of diabetic animals with 17β-oestradiol also protects pancreatic β cells against oxidative stress, amyloid polypeptide toxicity, lipotoxicity, and apoptosis. Similarly, reduced testosterone level is related to obesity, insulin resistance, type 2 diabetes, heart disease, benign prostatic hypertrophy, and even prostate cancer. In the final stage of the steroidogenic cascade, testosterone is metabolized to oestradiol by P450 aromatase, in the cytoplasm of adipocytes, breast cells, endothelial cells, and prostate cells, to increase intracellular oestradiol concentration at the expense of testosterone. The stress, xeno-oestrogens, poor dietary choices, and reactive toxins up-regulate aromatase to increase intracellular oestradiol production. Thus, the raised intracellular oestradiol levels in men, promote these pathologies (Kumar et al., 2012).

The inhibition of succination of adipocyte protein would be helpful in managing cellular stress. It can be achieved by using un-couplers of oxidative phosphorylation and by inhibitors of ER stress. Since, ER involves the release of cellular Ca^++^, which leads to activation of calpain and caspase-12 and cleavage of fodrin, so use of Bapta-AM (a cell permeable Ca^++^ specific chelator), or calpeptin (a calpain inhibitor) would prevent this ER stress and mediated apoptosis. The Ruthenium red, an inhibitor of mitochondrial Ca^++^, uniporter, and also prevents apoptosis by inhibiting the subsequent mitochondrial dysfunction. Similarly, Diospyrin diethylether (D7), a bisnaphthoquinonoid derivative, exhibits an oxidative stress-dependent apoptosis in several human cancer cells and tumor models (Fullwood et al., 2012).

The eIF2α phosphatases prolong eIF2α phosphorylation and thus reduce the cell death showing significant cyto-protection in several neurodegenerative disorders. Thus, it may be used as a potential strategy for future drug development to treat human protein mis-folding disorders (Jung et al., 2012). Similarly, Subtilase cytotoxin (SubAB), leads to ER stress and apoptosis by cleaving the molecular chaperone BiP in the ER. It is mediated by RNA-dependent protein kinase (PKR)-like ER kinase (PERK). Thus, treatment with proteasome inhibitors (i.e., MG132 and lactacystin) may be helpful. Further, Metformin, directly protects against dysfunction and death of ER stress-induced NIT-1 cells (a mouse pancreatic beta cell line) via AMP-activated protein kinase (AMPK) and phosphatidylinositol-3 (PI3) kinase activation (Isa et al., 2011).

Since NO-induced apoptosis in beta-cells of pancreas, is mediated by the ER-stress pathway by inducing CHOP (ER stress-associated apoptosis factor), so NO scavengers may be indirectly helpful in this mission. The antimycin or oligomycin are known to induce Metabolic stress in pancreatic β-cells. The impaired mitochondria dysfunction increases ER stress. Proteins such as p-eIF2α, GRP78 and GRP 94, CHOP activate JNK, and AMPK is involved in this process. Thus, its inhibition with any compound such as Compound-C, may block the ER stress and there by decrease the β-cell apoptosis. Similarly, those drugs which could reduce the cholesterol uptake and increase cholesterol efflux from macrophages, could suppress the ER stress and could be used as a potential therapy for atherosclerosis. In case of T-2 DM patients, there is predominance of the M1 over M2 phenotype in peripheral blood monocytes (Oh et al., 2012). Thus, suppression of ER stress would decrease the vascular inflammation and there by prevents the development of atherosclerosis (Bouhlel et al., 2009). Stimulation of PPARγ and PPARδ would also facilitate the transformation of undifferentiated macrophages to the alternatively activated M2 phenotype, there by inhibiting the activation of macrophage mediated inflammatory pathway (Tsukano et al., 2010). Since, ER stress also promotes insulin resistance, generating a vicious cycle that leads to plaque instability, so its suppression would be helpful in management of diabetes and vulnerable plaque management (McAlpine et al., 2010).

The use of herbal medicines is on continuous rise for management of life style related metabolic disorders. These preparation are being used both as medicine and also as food supplements. Most of these preparations possess antioxidant and anti-inflammatory property, due to presence of high poly phenolic compounds, so it would be logical to explore these plant extracts and also pure phytochemicals on management of ER stress. This part has not been fully exploited for novel drug development, but it has a great potential. Herbs like *Pueraria Tuberose* (Pandey et al., 2007), *Rubia Cordifolia* (Tripathi and Singh, 2007), *Vitex nigundo Linn*. (Tiwari and Triapthi, 2007), *Commiphora mukul* (Singh et al., 2005), Cinnamomum tamala Linn (Chaurasia and Tripathi, 2010), and *Nigella Sativa* (Tripathi et al., 2012) have already been reported to have strong antioxidant potential. They are able to trap the free radicals and also capable of chelating the metals.

## Conclusion

High nutritional load attracts induces ER stress and change the profile of chemokine release by adipocytes. These secretions, induce the phenotypic change in macrophages, which further add to the inflammatory process. The whole process leads to untimely death of overloaded adipocytes and macrophages, which further attracts the macrophages. Thus, prevention of ectopic and normal fat deposition would prevent the ER stress and mitochondrial dysfunction and associated apoptosis in these cells. Further low grade inflammation, induced by above cellular changes, could be prevented by reversal of the ER stress especially in adipose cells and macrophages. Many phytomolecules, which have reported antioxidant and anti-inflammatory property could be explored for their potential to reverse ER stress and associated inflammation/apoptosis, which may be finally useful for management of MS.

### Conflict of interest statement

The authors declare that the research was conducted in the absence of any commercial or financial relationships that could be construed as a potential conflict of interest.
